# *In Situ* Gas-Phase 4D-STEM for Strain Mapping during Hydride Formation in Palladium Nanocubes

**DOI:** 10.1021/acs.nanolett.5c00702

**Published:** 2025-03-25

**Authors:** Marta Perxés Perich, Jan-Willem Lankman, Claudia J. Keijzer, Jessi E. S. van der Hoeven

**Affiliations:** Materials Chemistry and Catalysis, Debye Institute for Nanomaterials Science, Utrecht University, 3584 CG Utrecht, The Netherlands

**Keywords:** 4D-STEM, lattice strain, *in situ* electron microscopy, hydride formation, palladium nanoparticles

## Abstract

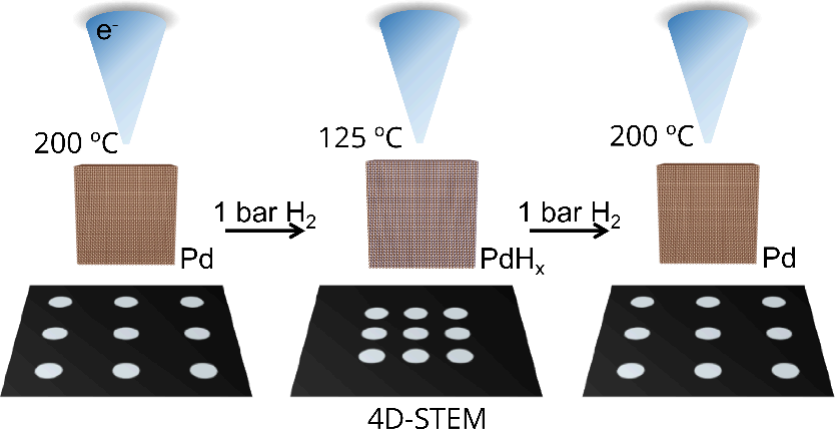

The uptake and release of hydrogen are key parameters for hydrogen storage materials. Lattice strain offers a powerful way to tune hydride formation in metal nanoparticles. However, the role of strain on hydride formation is difficult to assess on a single nanoparticle level due to the lack of *in situ* characterization tools to quantify strain in the presence of a gas. Here, we achieve a dynamic, *in situ* study on the reversible hydride formation in individual palladium nanocubes by applying 4D scanning transmission electron microscopy (4D-STEM) in the presence of 1 bar H_2_ and quantitatively assess the lattice strain with subnanometer resolution. Upon hydride formation at 125 °C, the Pd lattice expands by ∼3.1% and relaxes back upon hydrogen desorption at 200 °C. Our *in situ* 4D-STEM approach is relevant to a wide range of nanoparticle systems and applications, including catalyst- and gas-sensing materials.

The development of highly efficient hydrogen storage materials is necessary for the future use of hydrogen as energy carrier.^1^ Tuning the temperature and pressure of hydrogen absorption and desorption is critical in developing suitable materials for hydrogen storage applications. A common strategy to tune the hydrogen absorption and desorption equilibrium is to increase the surface to volume ratio of a metal by the formation of nanoparticles.^2−4^ Nanoparticle formation generally leads to enhanced hydrogen absorption kinetics with smaller particles showing a thermodynamic destabilization of the hydride phase.^2−5^ In addition to nanoscaling, lattice strain engineering can be used to tune the hydrogen absorption and desorption thermodynamics.^4,6,7^ Compressive lattice strain can destabilize the metal hydride phase,^4,7^ and has been used to tune the equilibrium hydrogen pressure in hydrogen storage materials over 5 orders of magnitude.^8^ However, it is often difficult to disentangle the effect of strain from other parameters like the nanoparticle size, shape, crystal structure and defects using ensemble averaged techniques.^9−11^

A particularly well-studied hydrogen storage material is nanostructured palladium (Pd). Palladium nanoparticles have extensively been used as model systems for understanding metal–hydrogen interactions and readily form a hydride phases at room temperature under hydrogen pressures below 1 bar.^12^ The structural control over the palladium nanoparticles has made them valuable materials for fundamental studies on the effect of nanoparticle size and shape on hydride formation, and revealed major differences between bulk and nanoscale.^13^ For example, ensemble measurements of Pd nanoparticles suggested a continuous phase transition from the hydrogen-poor α phase to the hydrogen-rich β-PdH_*x*_ phase,^14,15^ while single particle studies revealed that the phase transition is abrupt in single-crystalline nanoparticles.^13,16^ The dynamics of the α to β-PdH_*x*_ phase transition have been studied *in situ* using environmental transmission electron microscopy (TEM),^17^ which allowed mapping of the α and β phases in individual nanoparticles with ∼ 3 nm resolution.^18,19^ While single-crystalline nanoparticles showed a homogeneous β-PdH_*x*_ distribution, twinned nanoparticles showed reduced hydrogen storage capacity in the central regions of the nanoparticle.^18^ This difference was attributed to compressive strain at the nanoparticle twins, which destabilizes the hydride phase. Yet, the strain could not be assessed locally, nor quantified.^20,21,20,21^ Coherent X-ray diffractive imaging (CDXI) has been used to map strain in 3D *in situ* for observing the nucleation and growth the β-PdH_*x*_ phase of >100 nm Pd nanoparticles.^20,21^ However, the spatial resolution was limited to 16 nm. To fully understand the role of strain on the hydride formation in nanoparticle systems, it is crucial to develop *in situ* methodologies to locally quantify strain in the presence of H_2_ gas, on a single-nanoparticle level and with subnanometer resolution.

A novel and quantitative technique to locally measure strain in nanoparticles is 4D-STEM. In 4D-STEM, a (sub)-nanometer electron probe is scanned over the sample to record the local diffraction pattern at each pixel, obtaining 4D data consisting of 2D diffraction patterns over a 2D grid of probe positions.^22,23^ These local diffraction patterns are then used for strain mapping, which is based on the inverse relationship between interatomic distance and diffraction disk spacing in the diffraction patterns. 4D-STEM strain mapping is well established for semiconductor materials,^24−27^ but there are also successful examples in nanoparticles.^23,28^ However, 4D-STEM has predominantly been used under vacuum conditions so far. Yet, 4D-STEM is uniquely suited to assess strain variations under *in situ* gas phase conditions as well, because (i) it does not require atomic resolution, (ii) it is quantitative, (iii) it can be done at low electron dose, and (iv) it provides subnanometer resolved strain maps.^23,24^ These are clear advantages with respect to the current state-of-art *in situ* electron microscopy techniques, in which high-quality atomic resolution images are either analyzed by geometrical phase analysis^29,30^ or by detecting the position of atomic columns in 2D^31,32^ or 3D^33^ images. There are few examples of using 4D-STEM during *in situ* measurements, for strain mapping during *in situ* mechanical testing,^34,35^ for orientation mapping in the liquid phase,^36^ and under electrochemical potentials.^37,38^ However, while promising, the use of 4D-STEM has not been reported for gas-phase electron microscopy, nor has it been applied for *in situ* strain analyses of nanomaterials in the presence of reactive gases.

In this work, we demonstrate that *in situ* gas phase 4D-STEM is a powerful tool to quantify strain in single metal nanoparticles upon lattice expansion and contraction during hydrogen uptake and release. As a model system, we apply our methodology to well-defined palladium nanocubes and quantify the changes in lattice strain with subnanometer precision during the temperature-dependent hydride formation on a single-nanoparticle level. Our 4D-STEM measurements are conducted in the presence of 1 bar H_2_ gas. To the best of our knowledge, this is the first time that 4D-STEM strain measurements under atmospheric pressure are reported.

We used single crystalline ∼20 nm Pd nanocubes terminated by {100} planes synthesized through colloidal synthesis.^39^ High-angle annular dark-field scanning transmission electron microscopy (HAADF-STEM) images of the resulting nanocubes are displayed in Figure 1a–c. The high-resolution HAADF-STEM images were used to determine the lattice parameter, which was of 391 ± 2 pm, very similar to the 389 pm of bulk Pd. A typical nanobeam electron diffraction pattern (NBED) of a Pd nanocube oriented in the [001] zone axis under vacuum is shown in Figure 1d. The central disk in the NBED has the highest intensity and originates from nondiffracted electrons. The 200 and 020 diffraction disks are perpendicular to the surface of the nanocube and are marked by the green arrows in the NBED, indicating the x and y direction used in the strain calculations. The strain calculation is based on locating the center of the diffraction disks, and therefore it is important to avoid overlap between the disks. To achieve this, a small convergence angle of 2.5 mrad was used. Note that the low convergence angle lowers the spatial resolution as the probe size increases from ∼50 pm to ∼480 pm when decreasing the convergence angle from 20.0 mrad, used in the HR-STEM images (Figure 1a–c), to 2.5 mrad used for NBED acquisition (Figure 1d).

**Figure 1:**
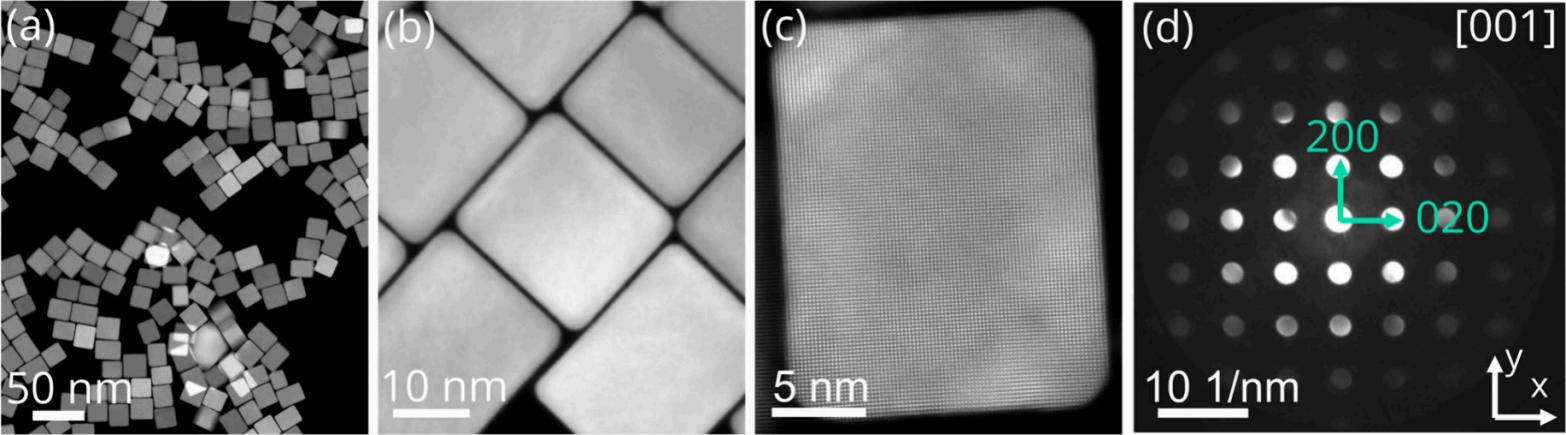
Colloidally synthesized Pd nanocubes. (a) Low- and (b) high-magnification High angular annular dark field scanning transmission electron microscopy (HAADF-STEM) images of as-synthesized Pd nanocubes of 19.7 ± 1.6 nm. (c) Atomic resolution HAADF-STEM image of a Pd nanocube. (a), (b) and (c) were obtained with a convergence angle of 20.0 mrad. (d) A typical nanobeam electron diffraction (NBED) pattern of a Pd nanocube oriented along the [100] zone axis obtained with a convergence angle of 2.5 mrad. The 200 and 020 diffraction disks correspond to the x and y direction used for the strain calculations. This specific NBED pattern originates from integrating all the 81 NBED patterns of a 3.6 × 3.6 nm region in the center of a Pd nanocube.

First, 4D-STEM was applied for quantitative strain mapping under *ex situ* conditions, in vacuum, after drop-casting the Pd nanocubes onto a TEM grid and removing the ligands with activated carbon (see Methods).^40^ The NBED patterns were acquired with a step size of 0.4 nm, which is slightly smaller than the electron probe. The subsequent analysis of these patterns was performed using Gatan Microscopy Suite environment.^41^ In short, the software calculates strain by measuring the distances between the central disk and the collinear diffraction disks (up to the second diffraction order) for every pixel, and compares these distances to those in the NBED patterns of a reference region.^42^ This analysis is performed in the two directions perpendicular to the nanocube surface, corresponding to the 200 and 020 diffraction disks marked in Figure 1d, and yields the ε_*xx*_ and ε_*yy*_ strain maps, which indicate the extension or contraction of the lattice in the x and y direction, respectively. Further details on the strain mapping acquisition and analysis are provided in the Methods section in the Supporting Information.

We used the center of each nanoparticle as a strain-free reference area, and calculated the strain in the rest of that corresponding nanoparticle relative to the averaged NBED pattern of the reference area. Moving the reference area to different regions in the nanoparticle interior has little effect in the final strain map (Figure S1), unless it is placed in a strained region. A reference area of 3.6 × 3.6 nm (9 × 9 pixels) was large enough to remove noise and small artifacts from a NBED pattern coming from a single pixel. At the nanoparticle surface (≤1 nm from the edge), the NBED patterns were slightly deformed leading to artifacts in the strain map (Figure S2). Therefore, those pixels were manually removed from the final strain map. Similar artifacts in the NBED pattern have been observed at sharp interfaces between regions with an abrupt change of lattice parameter,^25^ and are probably caused by the relatively large probe size that simultaneously probes the Pd cubes and the vacuum. This issue could be solved by using electron beam precession, which allows the use of larger convergence angles, thereby forming smaller probes.^43−45^ Patterned probes have also been suggested as a strategy to further increase the strain mapping precision,^46^ although they might not solve the artifacts at the nanoparticle edges.^44^

In vacuum, the Pd nanocubes exhibit tensile strain at the nanoparticle edges in all analyzed cases. The reference area for each nanoparticle is indicated with the gray box in the ADF images of Figure 2a, with the corresponding integrated NBED patterns shown in Figure 2b. The ε_*xx*_ and ε_*yy*_ strain maps are shown in Figure 2c and 2d, respectively. Strain in the nanoparticle bulk stayed below 0.5% relative to the reference, while strain at the edges varied between 1% and 1.5%. This was further corroborated by the line profiles shown in Figure S3, which also show that strain is usually larger in the out-of-plane direction. Our data are in line with previously reported strain profiles of Pd nanoctahedra,^47^ and other nanoparticle systems.^32,33,48^ It is unlikely that the observed tensile strain originates from surface oxidation (Figure S4), which is more prominent in smaller Pd nanoparticles.^5^

**Figure 2:**
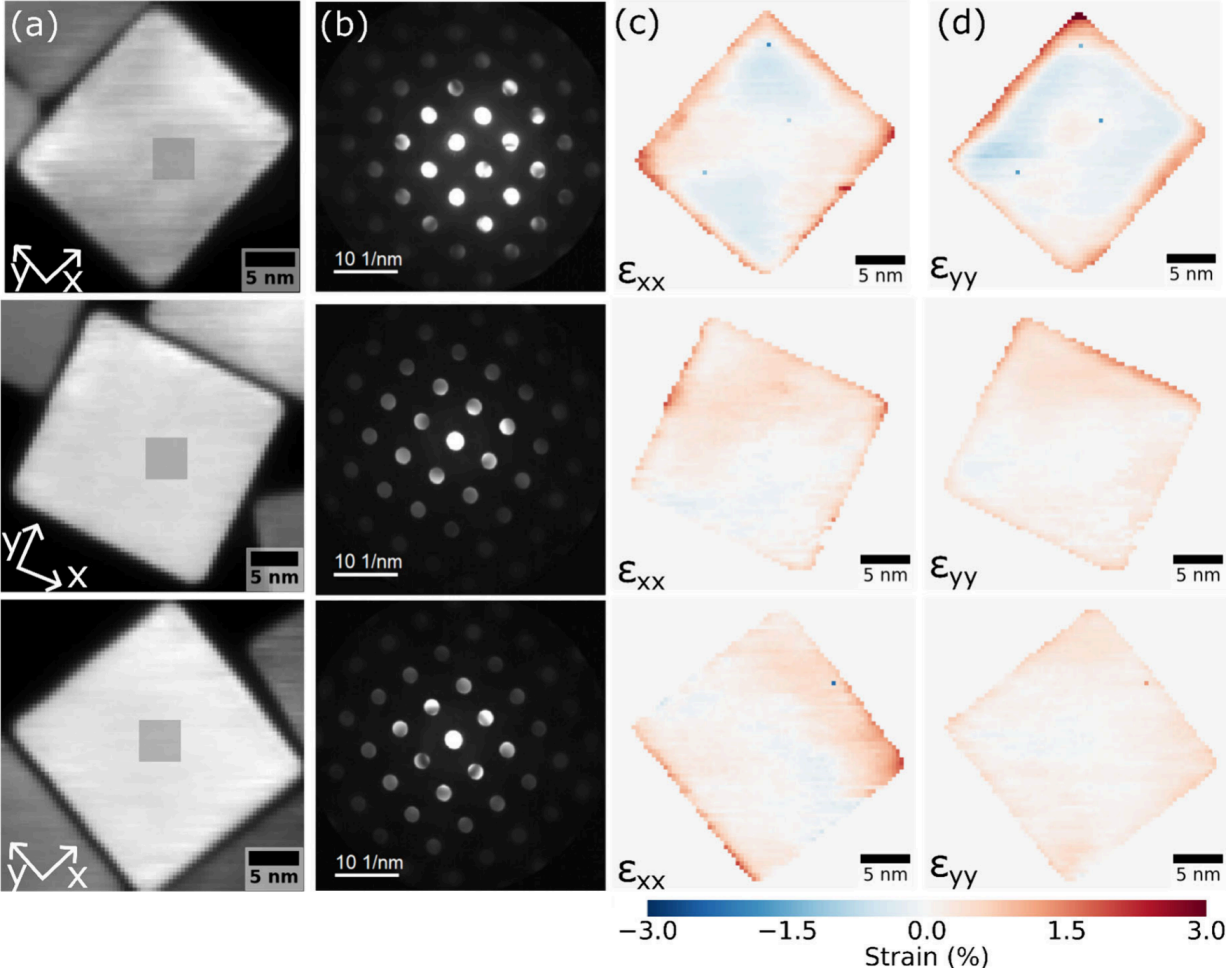
Strain mapping with 4D-STEM of three different Pd nanocubes at room temperature in vacuum. (a) Annular dark field (ADF) images. Each pixel contains a NBED pattern. The shadowed regions are used as reference regions for strain mapping in the corresponding particle. (b) NBED patterns of the reference area (3.6 × 3.6 nm, 9 × 9 pixels), rotated −86 ° from the ADF images. Note that all analyzed particles are oriented in the [001] zone axis and show up to the 3rd diffraction order in the NBED pattern. (c) Strain maps in the *x* (ε_*xx*_) and (d) *y* (ε_*yy*_) direction for each nanoparticle. The color scale is shown at the bottom with red and blue corresponding to tensile and compressive strain, respectively. A mask was applied to remove the pixels that are not coming from the nanoparticle of interest and the pixels with distorted NBED patterns within the first 1 nm from the surface.

Upon introducing a gas pressure of 1 bar H_2_, the NBED patterns were barely affected and remained of excellent quality. This was in large part due to the usage of an energy filter with a slit centered in the zero-loss peak that only allowed the elastically scattered electrons to reach the CCD camera to form the NBED pattern, leading to a high signal-to-noise NBED pattern with sharp diffraction disk edges (Figure 3a). In contrast, without the energy filter the edges of the diffraction disks were blurry, particularly in the central disk (Figure 3b). Maintaining a high signal-to-noise ratio and sufficient sharpness of the diffraction disk edges is crucial for the strain mapping algorithm to precisely locate the center of the diffraction disks and subsequently calculate the strain (Figure S5).^42^Figure 3c and d shows energy-filtered NBED patterns obtained under H_2_ and Ar of the same region, and Figure S6 also includes a NBED pattern under O_2_. The diffraction disks always show sharp edges, but in some cases the signal-to-noise ratio was slightly lower in the presence of 1 bar Ar or O_2_ gas, possibly due to more electron scattering caused by higher Z number of argon compared to hydrogen. Therefore, the use of an energy filter is preferred for 4D-STEM gas-cell experiments, and allows obtaining high-quality NBED patterns under various gas atmospheres, with higher signal-to-noise ratios.

**Figure 3:**
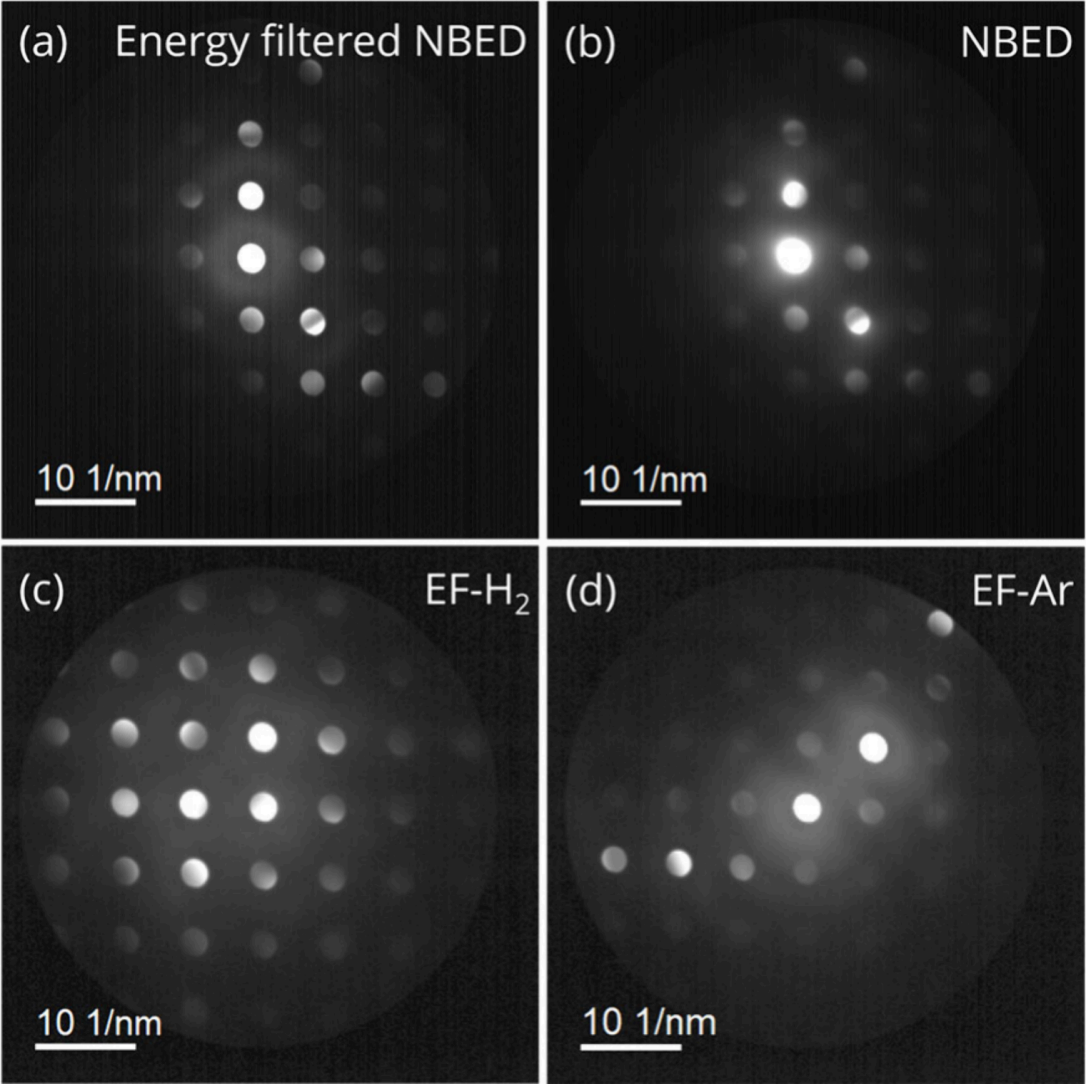
Effect of the energy filter and the gas atmosphere in the NBED pattern. (a, b) NBED patterns of the same area under 1 bar H_2_ (a) with and (b) without applying the 5 eV energy filter (EF). (c) and (d) EF-NBED patterns of the same area under 1 bar (c) H_2_ and (d) Ar atmosphere.

Next, we performed *in situ* 4D-STEM under 1 bar H_2_ flow to observe the lattice expansion of Pd nanoparticles during hydrogen absorption and release upon cooling and reheating. The temperature and gas profile are displayed in Figure S7. First, the sample was dried with a 30 min pretreatment in a 1 bar Ar flow at 200 °C. Next, the gas was switched to a flow of 1 bar H_2_, and the temperature was adjusted from 200 °C to 90, 125, 150, 175, and 200 °C. At each temperature, 4D-STEM data sets of several nanoparticles were collected, with each acquisition taking ∼2 min. To guarantee that the 4D-STEM measurements were taken under equilibrium conditions, we let the system stabilize at each temperature for at least 5 min before starting the measurement, which is much longer than the 20 s in which the α to β-PdH_*x*_ transition had been observed for ∼20 nm nanocubes at −27 °C and 283 Pa.^17^ The stabilization period also helped avoiding the drift artifacts caused by the stretching of the silicon nitride membrane of the chips at different temperatures.

We successfully acquired *in situ* 4D-STEM data sets of four individual Pd nanocubes, monitored over the range of temperatures. The nanoparticle orientation did not significantly change during the measurements at different temperatures and upon electron beam exposure, and remained close to zone axis throughout the experiment (Figure S8). The robustness of 4D-STEM strain mapping toward small deviations of the zone axis^23,24^ allowed using the same reference throughout the range of temperatures. We chose to use the nanoparticle at 200 °C as reference, where little hydrogen uptake is expected.^49^ This reference area is marked as a gray square overlaid on the strain maps in Figure 4a.

**Figure 4:**
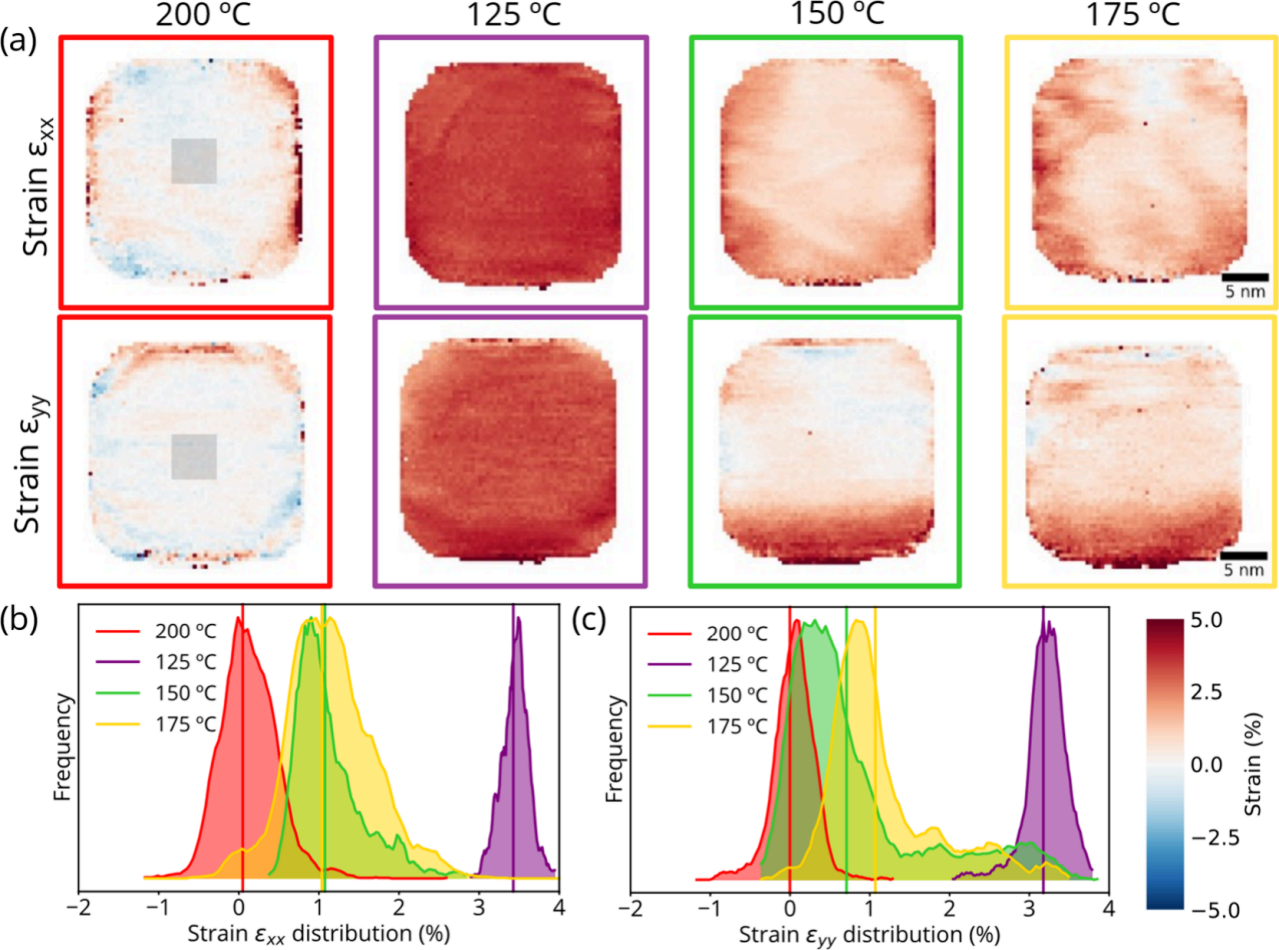
Strain mapping of a Pd nanocube at 1 bar H_2_. (a) Strain maps (*x* and *y* directions) obtained from 4D-STEM data sets using a region in the nanoparticle at 200 °C as reference (overlaid gray square in the 200 °C strain maps). The color scale is shown at the bottom with red and blue corresponding to tensile and compressive strain, respectively. (b) and (c) Normalized histogram of the strain distribution within a 10 × 10 nm region in the center of the nanoparticle at each temperature for strain in the x and y direction, respectively. The vertical lines correspond to the average strain at each temperature.

Figure 4a shows the 4D-STEM strain maps for a Pd nanoparticle under constant hydrogen flow at 1 bar with lattice expansion upon hydrogen uptake at 125 °C and lattice contraction upon H_2_ release between 150 and 200 °C. The results of three additional nanoparticles are shown in Figures S9–S12 and also include the strain map upon return to 200 °C. The initial strain maps at 200 °C (Figure 4a) are very similar to those obtained under vacuum (Figure 2), with ∼1.5% tensile strain close to the nanoparticle surface. Some morphological changes of the Pd NPs occurred upon heating to 200 °C during the pretreatment step in Ar, such as rounding of the nanocube edges, merging of nanoparticles in close proximity (Figures S10–S12) and partial loss of the cubic shape (Figure S11). The more rounded particles exhibited less tensile strain at the nanoparticle surface, possibly due to partial surface relaxation. The nanoparticle shape stayed constant throughout the rest of the experiment in H_2_.

Upon hydrogen absorption at 125 °C, the lattice of the Pd nanoparticles expanded homogeneously by 3.1 ± 0.3% on average. The nanoparticle shape did not change upon hydrogen intake. The strain distribution within the nanoparticles is illustrated in the histograms in Figures 4b and 4c. The average strain at 125 °C in the nanoparticles ranged from 2.7 and 3.4%. This is slightly lower than the 3.5% lattice expansion measured for similar nanocubes at −35 °C and 500 Pa,^17^ in line with the known destabilization of the hydride phase with increasing temperature.^14^ Using the linear relation between the lattice parameter and the H/Pd ratio in the β-PdH_*x*_ phase,^50^ we calculated that the H/Pd ratio was 0.58 ± 0.07 (further details of the calculation in the Supporting Information, Supplementary Note 1). Homogeneous hydrogen uptake is expected for single-crystalline nanoparticles upon the formation of the β-PdH_*x*_ phase,^18^ while an inhomogeneous hydrogen distribution is more likely to occur in much larger ∼100 nm nanocubes.^20^ Our subnanometer resolution 4D-STEM strain mapping results reveal that there is no difference in H composition between the surface and bulk for 20 nm Pd nanocubes.

Hydrogen desorption (β to α-PdH_*x*_ phase transition) happened between 125 and 150 °C, as observed by the lattice contraction at 150 °C and no further significant changes upon reheating to 200 °C. This is in line with literature, where 8 nm nanoparticles only showed H_2_ absorption up to 120 °C.^14,51^ The strain maps upon desorption show a more heterogeneous hydrogen distribution, but without clear trends of hydrogen accumulation at certain location (Figure 4 and Figures S9–S12). Our results do not show a clear core–shell^16^ or spherical-cap distribution of a H-rich phase and a H-poor core as reported previously for H absorption in Pd nanoparticles.^17,21^ The inhomogeneous strain distribution that we observe upon desorption could be ascribed to local hydrogen trapping into the Pd lattice, although this more likely to occur in smaller (<6 nm) nanoparticles.^52^

Hydrogen chemisorption experiments confirmed that the Pd nanocubes did not absorb hydrogen at 200 °C, indicating that the reference used in the 4D-STEM data sets is close to non-hydrogenated Pd. The isotherms (Figure S13a) revealed that the maximum quantity of absorbed hydrogen decreased with increasing temperature while simultaneously the pressure required for hydrogen absorption increased, which is in line with literature.^14^ Pressure–composition isotherms derived from the hydrogen chemisorption data (Figure S13b) show the evolution of the H/Pd ratio with increasing H_2_ pressure, and the flat plateau pressure, corresponding to the α- to β-PdH_*x*_ transition, indicating bulk-like behavior.^12^ The hydrogen content in the β-PdH_*x*_ phase decreased from PdH_0.7_ at 30 °C to PdH_0.66_ at 90 °C. The value expected from the 4D-STEM data is PdH_0.58_, slightly lower than the value at 90 °C, which follows the trend of lower H_2_ solubility with increasing temperature. These compositional changes are consistent with the smaller lattice expansion (3.1%) observed with 4D-STEM at 125 °C, compared to the 3.5% expansion reported in prior room-temperature studies.^17^

Interestingly, the lattice parameter did not fully return to its original value in our 4D-STEM experiments, and a slight lattice expansion of 0.5 ± 0.7% with respect to the nanoparticle at 200 °C prior to H_2_ absorption upon return to 200 °C This could be due to trapping of a small amount of hydrogen,^52^ which was undetected in our chemisorption measurements. Alternatively, the measurement itself could have caused the apparent lattice expansion upon reheating to 200 °C, for instance due to carbon build-up, which reduced the signal-to-noise ratio in the NBED patterns, resulting in noisier strain maps (Figure S12). To check whether hydrogen trapping or carbon contamination was the main cause for the incomplete return to the original lattice, we conducted an extra experiment with less beam exposure and additional heating steps to 250 and 300 °C (Figures S14–S15). No carbon contamination was observed during the experiment. Figure S16 shows a comparison of the strain values obtained in the two experiments. Again, homogeneous 3.1 ± 0.2% strain was observed at 125 °C, exactly reproducing the results of our previous experiment in a new mounted gas-cell. Upon reheating to 200 °C, only an average 0.1 ± 0.4% strain remained, indicating higher (but not complete) recuperation of the Pd lattice. The lattice further contracted when heating at 250 °C (−0.1 ± 0.5%) and 300 °C (−0.7 ± 0.3%). This suggests that the nanoparticles at 200 °C still contained some hydrogen in their lattice, in line with the α-PdH_0.025_ composition found in literature at these conditions^49^ and the PdH_0.03_ composition calculated from the 0.7% lattice expansion with respect to the hydrogen-free nanocube at 300 °C.^50^

To exclude any temperature induced effects on the lattice strain, a separate heating experiment in vacuum was performed, collecting 4D-STEM data sets with the same temperature profile. Indeed, no clear lattice expansion or contraction was observed when changing the temperature between 25 and 200 °C without the presence of H_2_ (Figure S17). As in the gas-cell experiments, the nanoparticle at 200 °C was used as the reference, and the lattice expansion relative to it remained close to 0.0 ± 0.2%. The thermal lattice expansion of Pd is expected to be below <0.1% from 125 to 200 °C and ∼0.2% between 25 and 200 °C.^53^ These values are close to the error of our measurement. This confirmed that the 3.1 ± 0.3% lattice expansion observed under H_2_ at 125 °C with respect to the lattice at 200 °C was completely caused by the uptake of hydrogen in the Pd lattice.

In conclusion, we were able to follow the hydrogen absorption and desorption in Pd nanoparticles under 1 bar H_2_ using 4D-STEM strain mapping. We first showed that pristine Pd nanocubes have ∼1.5% tensile strain at the nanoparticle edges, which is less apparent after heat treatment and in the presence of a gas atmosphere. Then, we quantified the lattice strain upon hydrogen absorption in the Pd lattice in 1 bar H_2_. The observed homogeneous ∼3.1% lattice expansion at 125 °C measured in our *in situ* experiments with respect to the lattice at 200 °C is in line with ensemble hydrogen chemisorption experiments. All in all, we believe that the development of *in situ* 4D-STEM strain mapping under a gas atmosphere will not only be relevant for hydrogen storage materials but also for a wide range of nanoparticle systems, including nanoparticle catalysts under working conditions and semiconductors.

## Data Availability

The three 4D-STEM data sets under vacuum and gas-phase 4D-STEM measurements for one nanoparticle are available at DataverseNL upon publication (reference^54^). Due to the large volume of the 4D-STEM data sets (∼5 GB per file), the rest of the data is available from the authors upon request.

## Supporting Information Available

Methodology section and supporting figures (PDF)
