# Supplementary material for: In Situ Gas-Phase 4D-STEM for Strain Mapping during Hydride Formation in Palladium Nanocubes
Source: Nano Lett. 2025 Mar 25;25(13):5444–51. doi: 10.1021/acs.nanolett.5c00702 (PMC11969644; doi:10.1021/acs.nanolett.5c00702)
Supplement: Supplementary file 1 — nl5c00702_si_001.pdf [file nl5c00702_si_001.pdf]

Supplementary information: *In Situ* Gas-Phase 4D-STEM for Strain Mapping during Hydride Formation in Palladium Nanocubes

Marta Perxés Perich[1], Jan-Willem Lankman[1], Claudia J. Keijzer[1], Jessi E.S. van der Hoeven[1*]

[1] *Materials Chemistry and Catalysis, Debye Institute for Nanomaterials Science, Utrecht University, 3584 CG Utrecht, The Netherlands*

*E-mail: j.e.s.vanderhoeven@uu.nl

Table of Contents:

Methods....2

Synthesis of Pd nanocubes....2

Deposition on a silica support....2

Hydrogen chemisorption....2

4D scanning transmission electron microscopy....2

In situ gas-phase 4D-STEM....3

In situ heating transmission electron microscopy (under vacuum)....3

Supplementary Figures 1-16....4

Supplementary note 1....14

References....15

Methods

Synthesis of Pd nanocubes

All chemicals were purchased from Merck unless stated otherwise. Pd nanocubes were synthesized using a previously described procedure.[1,2] Briefly, 205 mg of PVP (M_w = 55.000), 125 mg of ascorbic acid, 1.223 g of KBr and 16.0 mL of deionized water (18.2 MΩ·cm) were heated to 80 °C for 10 min under magnetic stirring. Then, 113.2 mg of Na_2PdCl_4 (98% purity) dissolved in 6.0 mL of water were introduced into the pre-heated solution. The vial was capped and maintained at 80 °C for additional 3 h. The Pd cubes were washed by centrifugation (1x H_2O, 2x ethanol) and stored in 30 mL EtOH. The size distribution was measured by TEM by measuring the diagonal of the Pd nanocubes.

Deposition on a silica support

700 mg of silica (Aerosil OX50, Degussa) were mixed with 10.0 mL EtOH and sonicated for 15 minutes to ensure proper dispersion. 24 mL of Pd nanocubes from synthesis was centrifuged and redispersed in 20.0 mL EtOH and added to the silica suspension. After sonicating for approximately one hour, 15.0 mL of toluene was added to the suspension, and the mixture was sonicated for extra 5 minutes to ensure homogeneous deposition on the SiO_2 support. Then, it was centrifuged at 2000 rcf for 5 minutes and the clear supernatant was removed. The pellet was dried in an oil bath at 60 °C overnight, and sieved to a 90-212 µm fraction. To remove the ligands, the supported Pd/SiO_2 nanoparticles were heated in a U-shaped reactor under a 200 mL/min flow. First they were heated to 400 °C with a ramp of 5 °C/min under N_2 for two hours, and then they were kept at 250 °C under 20% O_2 in N_2 for two hours extra. This resulted in a 3.99 wt% Pd on the Aerosil OX50 support, as measured by ICP (Mikroanalytisches Laboratorium Kolbe).

Hydrogen chemisorption

Hydrogen chemisorption was measured using a Micrometrics ASAP 2020 analyzer. 200-300 mg of sample was loaded in a U-shaped quartz reactor between two layers of quartz wool. Before the measurement series, hydrogen was flown for 1 h at 250 ºC and subsequently evacuated. Hydrogen isotherms were measured between 0.001 to 1 bar at a range of temperatures, from 30 to 200 ºC, while evacuating at 100 ºC between measurements. The equilibration time at each temperature was of 10 s.

4D scanning transmission electron microscopy

Scanning transmission electron microscopy (STEM) was performed at a Thermofisher Spectra 300 operated in STEM mode at 300 kV. The Pd nanocubes were dropcasted onto a carbon coated TEM grid or a SiN chip, and the PVP ligands were removed by washing the grid or chip with activated carbon. In short, in a small beaker, activated carbon was mixed with ethanol. When the bubbling stopped, the grid or chip was submerged for 5 minutes and then let dry.[3]

One 4D-STEM dataset consisted of an annular dark field (ADF) image with a nanobeam electron diffraction (NBED) pattern at each pixel. The convergence angle was set to 2.5 mrad to ensure non-overlapping diffraction disks, and a camera length of 29 mm allowed observation of three orders of diffraction. NBED patterns were collected in a CCD camera placed after the Gatan Continuum energy filter, which was operated with a slit of 5 eV centered on the zero loss peak. 4D-STEM datasets were acquired with a step of 0.4 nm, 0.2 s of exposure time and 512x512 pixels in the CCD camera. The beam current was ~30 pA. A typical 4D-STEM dataset of a ~20 nm Pd nanocube was acquired in 2 minutes and had a size of 5 gigabytes.

The strain maps were obtained using the Gatan Digital Micrograph strain mapping module.[4] Prior to analysis, the central disk was aligned among the dataset. A 3.5x3.5 nm reference area was defined in the center of each nanoparticle. The strain was measured in the 100 and 010 directions, which are perpendicular to the nanoparticle edges, by selecting the 200 and 020 diffraction disks, as represented in Figure 1d. Strain was calculated by measuring the change in distance from the central disk and the colinear disks up to the 2nd diffraction order at each pixel. This was done in the x and y direction, and produced the ε_{xx} and ε_{yy} strain maps, respectively. For each nanoparticle, we performed the strain mapping calculation using at least two reference areas to ensure that the strain map does not significantly change after using two different regions as reference. The -86 ° rotation between the acquired NBED patterns and the ADF images was applied in the software to provide understanding on the strain direction. The NBED pattern at ~1 nm from the NP surface is typically deformed (Figure S2c), which produces artifacts in the strain map. Those pixels were removed by making a mask at ImageJ, making sure that only the pixels without artifacts in the NBED patterns were used for the final strain maps.

High resolution images were obtained with a convergence angle of 20.6 mrad, 145 mm camera length, a screen current of 94 pA and a pixel size of 27.29 pm. 20 images acquired with a 0.5 µs dwell time per pixel were combined through DCFI algorithm in the Velox software.

In situ gas-phase 4D-STEM

The *in situ* gas-phase STEM measurements were performed using a Protochips Atmosphere system and STEM/EDX optimized chips. The holder was pump-purged 10 times to 1 mbar to remove air from the system. All gasses (Linde, 6N purity) were introduced at atmospheric pressure and 0.1 sccm flow rate. The sample was dried at 200 °C in Ar for 30 minutes, and the experiment consisted of switching to 100% H_2 flow at 200 °C and varying the temperature to 90, 125, 150, 175 and 200 °C, as shown in the temperature profile in Figure S5. On a separate experiment, the temperature was varied from 200 °C to 90, 125, 200, 250 and 300 °C. 4D-STEM datasets of several nanoparticles of interest were recorded at each temperature. At each temperature the sample was allowed to stabilize for >5 min to reach steady state and to avoid sample drift during 4D-STEM mapping. Imaging at 90 °C resulted in significant contamination, possibly due to the presence of small amounts of water. In the second experiment, the beam was never on at 90 °C. This problem was solved by imaging above 100 °C, where water immediately evaporates.

In situ heating transmission electron microscopy (under vacuum)

The *in situ* heating TEM experiments in vacuum were performed using a DENS solutions Wildfire heating holder. The same temperature profile as in the gas-cell experiment was followed: a drying at 200 °C for 30 minutes followed by 4D-STEM maps at 200, 125, 150, 175,200and 25 °C of several nanoparticles.

Supplementary Figures 1-16

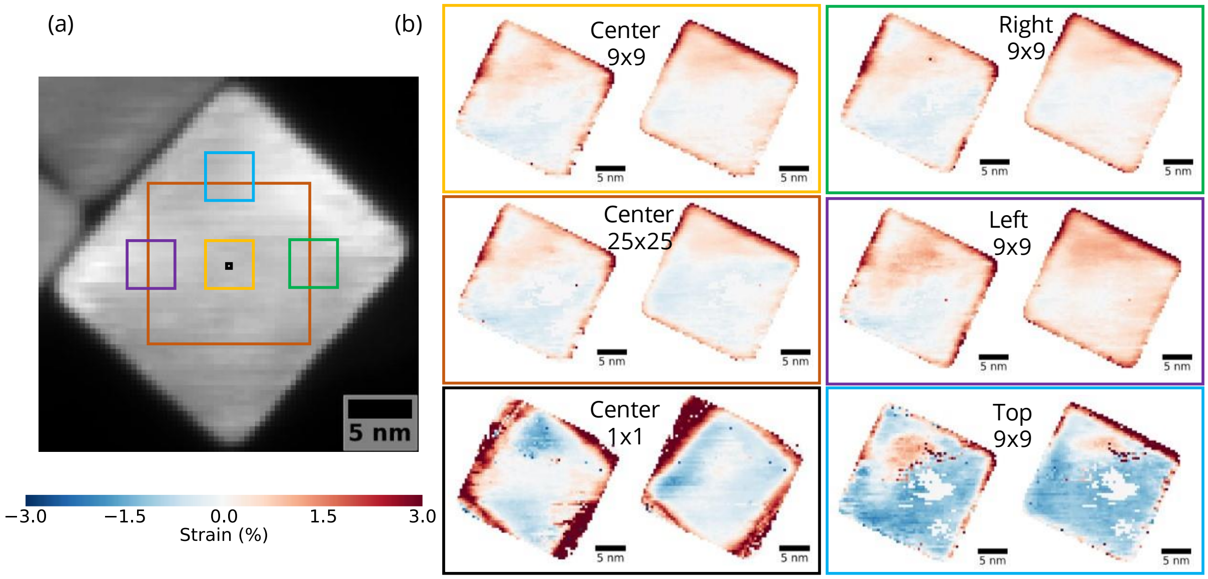

Figure S1. Strain maps obtained with different reference areas on the same nanoparticle. (a) ADF image with different reference areas overlaid. (b) Strain maps obtained using the reference area of the same color marked in (a). The reference areas had 9x9 pixels (3.6x3.6 nm), 25x25 pixels (10x10 nm) or 1x1 pixels (0.4x0.4 nm) of size. Scale of strain is ±3%. (see legend). Note that some tensile strain (~0.5%) strain is found in the top area of the nanoparticle. Therefore, when selecting this area as reference the results were different (bottom right panel indicated with the light blue box).

To obtain the final strain maps shown in the rest of the figures, a 9x9 pixel (3.6x3.6 nm) reference was placed in at least two different regions of the center of the nanoparticle. The results were compared, and if they were similar the reference was chosen to be at the center of the nanoparticle.

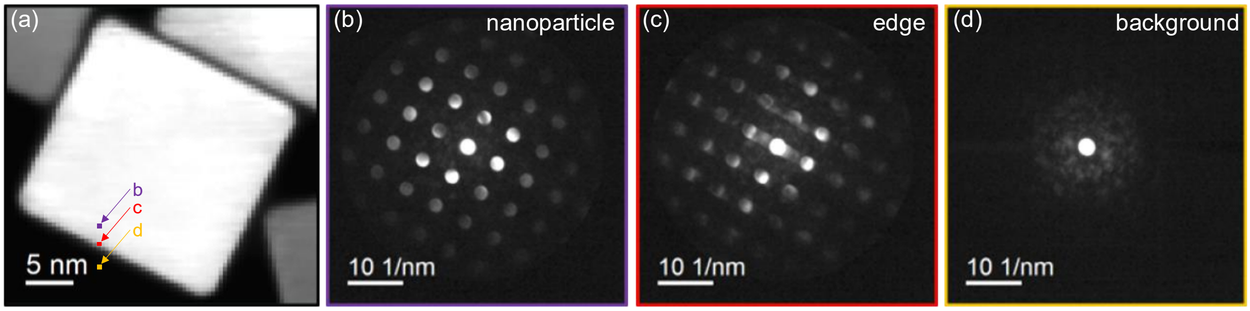

Figure S2. NBED patterns recorded at different locations in the nanoparticle. (a) ADF image with three signaled points corresponding to patterns in b (purple), c (red) and d (yellow). (b) Representative NBED pattern in the nanoparticle. (c) Representative NBED pattern at the nanoparticle edge. (d) Representative NBED pattern in the absence of nanoparticle: the central non-diffracted beam.

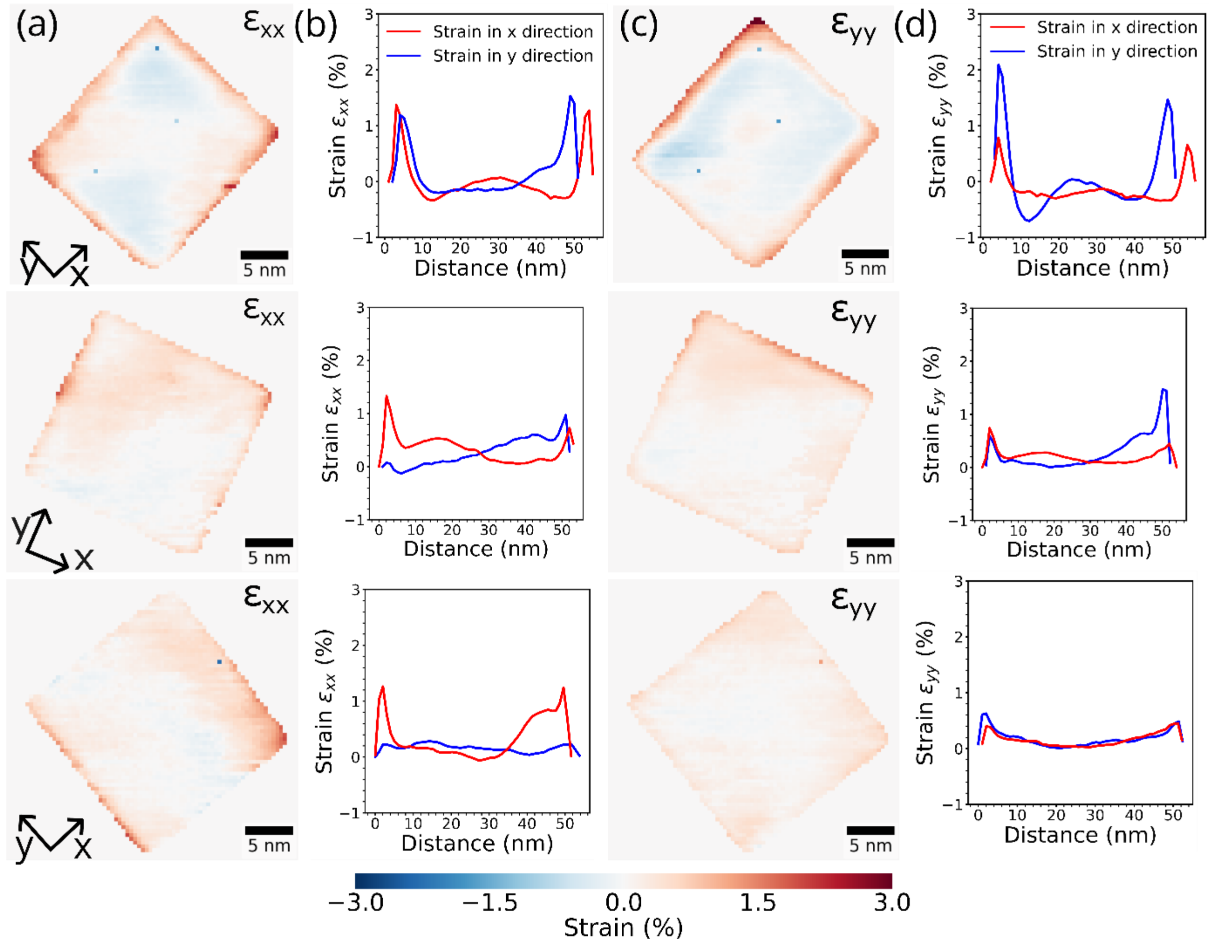

Figure S3. Line profiles of the strain maps of the Pd nanocubes in vacuum. The strain maps in (a) and (c) correspond to the strain ε_{xx} and ε_{yy} measurements shown in Figure 2c and 2d, respectively. (b) Line profiles of strain distribution in the x (red) and y (blue) direction in the strain ε_{xx} map. (d) Line profiles of strain distribution in the x (red) and y (blue) direction in the strain ε_{yy} map. The thickness of the line profile was of 20 pixels.

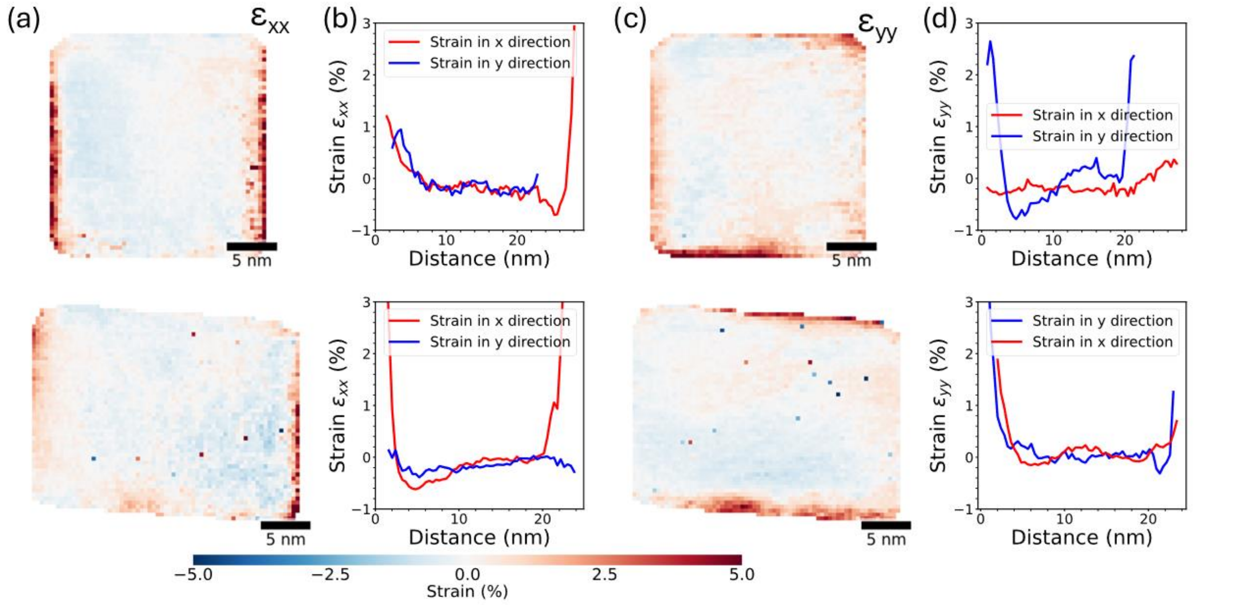

Figure S4. Strain mapping and line scans of two different nanoparticles after removal of a possible Pd oxide in vacuum after a 15 min, 30 mL/min H_2 treatment at room temperature followed by a vacuum treatment at 120 °C for 4 h and minimizing the exposure to air upon transferring the sample to the electron microscope.[5,6] The strain maps in (a) and (c) correspond to the strain ε_{xx} and ε_{yy} measurements of two different nanoparticles. (b) Line profiles of strain distribution in the x (red) and y (blue) direction in the strain ε_{xx} map. (d) Line profiles of strain distribution in the x (red) and y (blue) direction in the strain ε_{yy} map. The thickness of the line profile was of ~20 pixels.

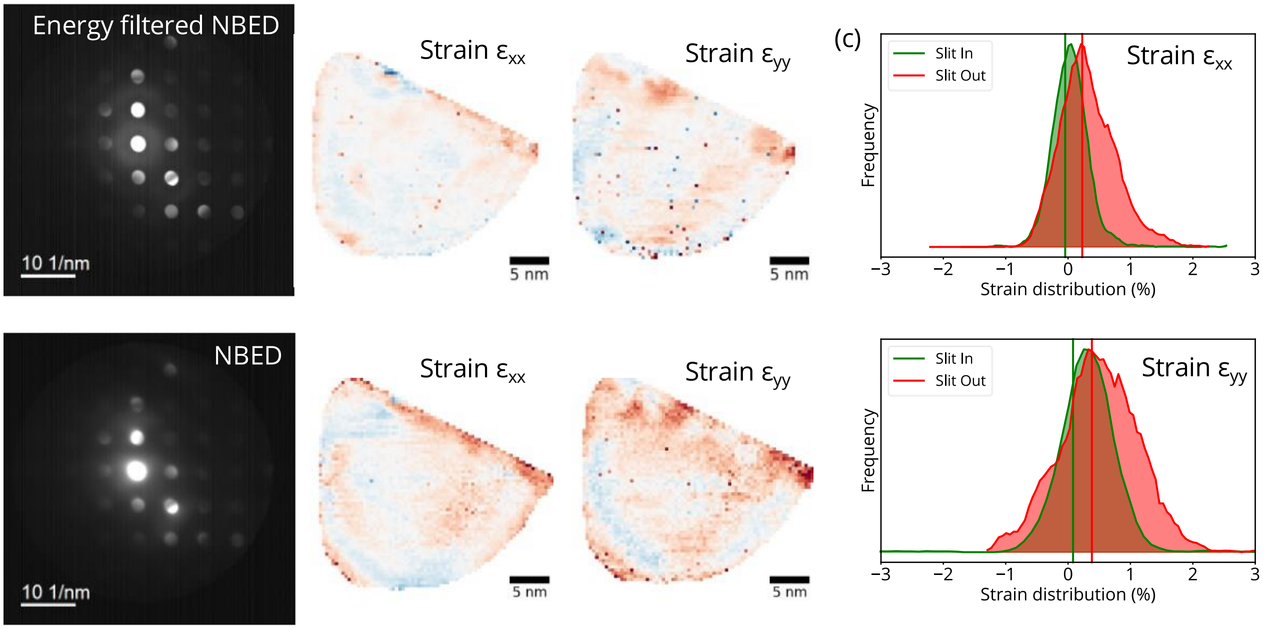

Figure S5. Comparison of strain maps obtained with (a) and without (b) energy filtering in the presence of 1 bar H_2. The data in a and b were recorded on the same nanoparticle. The strain maps obtained with the energy filter (a) show lower internal strain and more narrow strain distributions, as

demonstrated in the (c) strain histogram of a 14x14 nm region in the center of the nanoparticle of the strain ε_{xx} and ε_{yy} obtained with the energy filter (slit in, green) or without the energy filter (slit out, red).

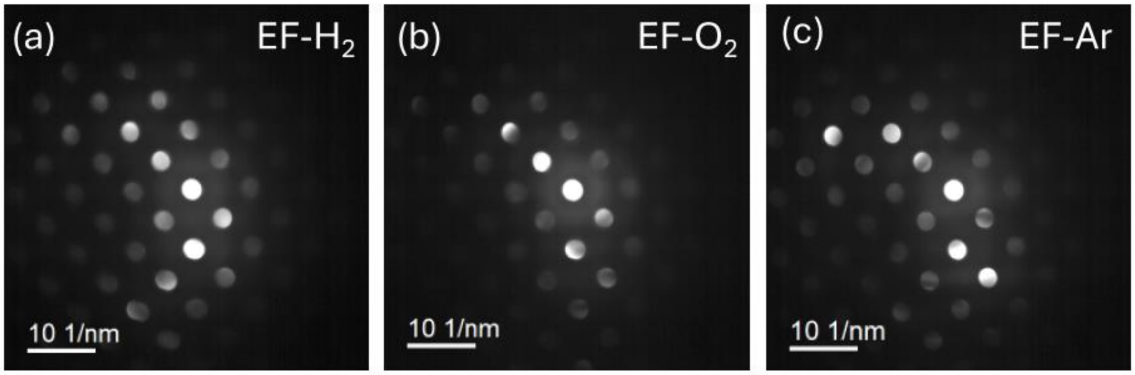

Figure S6. Effect of the gas on the energy-filtered NBED pattern. Energy-filtered NBED patterns of the same area in (a) H_2, (b) O_2, and (c) Ar, with the contrast adjusted in the same manner.

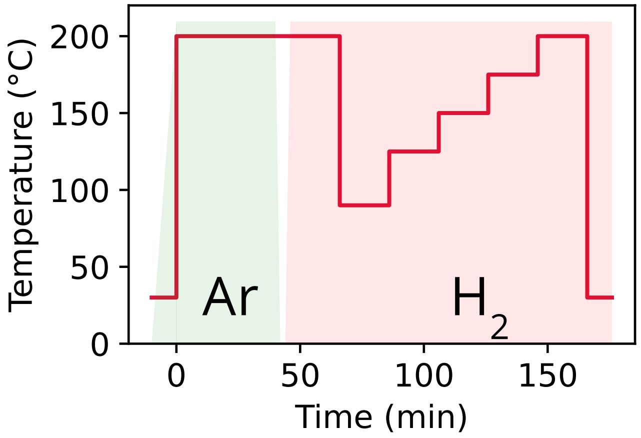

Figure S7. Example temperature and gas profile of an *in situ* experiment. All gasses were introduced with 1 bar pressure and a flow of 0.1 sccm. First, a pretreatment of 30 min at 200 °C under Ar was performed, followed by switching to H_2. Then, the temperature was modified from 200 °C to 90, 125, 150, 175 and 200 °C and 4D-STEM datasets of several nanoparticles were taken at each temperature.

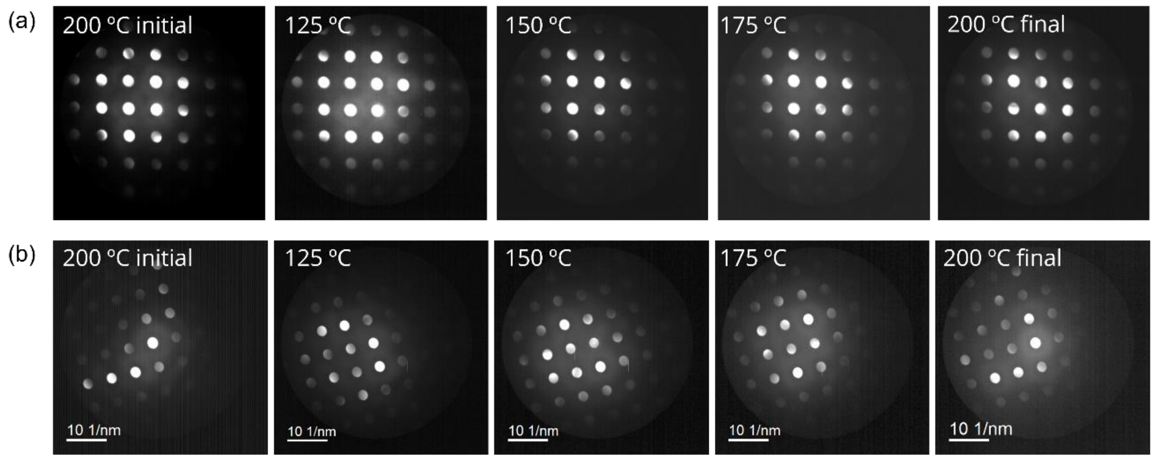

Figure S8. Change of orientation during the *in situ* experiment. Change of the averaged NBED pattern over 3.6x3.6 nm during the *in situ* gas-phase experiment at different temperatures following two different nanoparticles (a) NP shown in Figure S8 (b) NP shown in Figure 4 and S7.

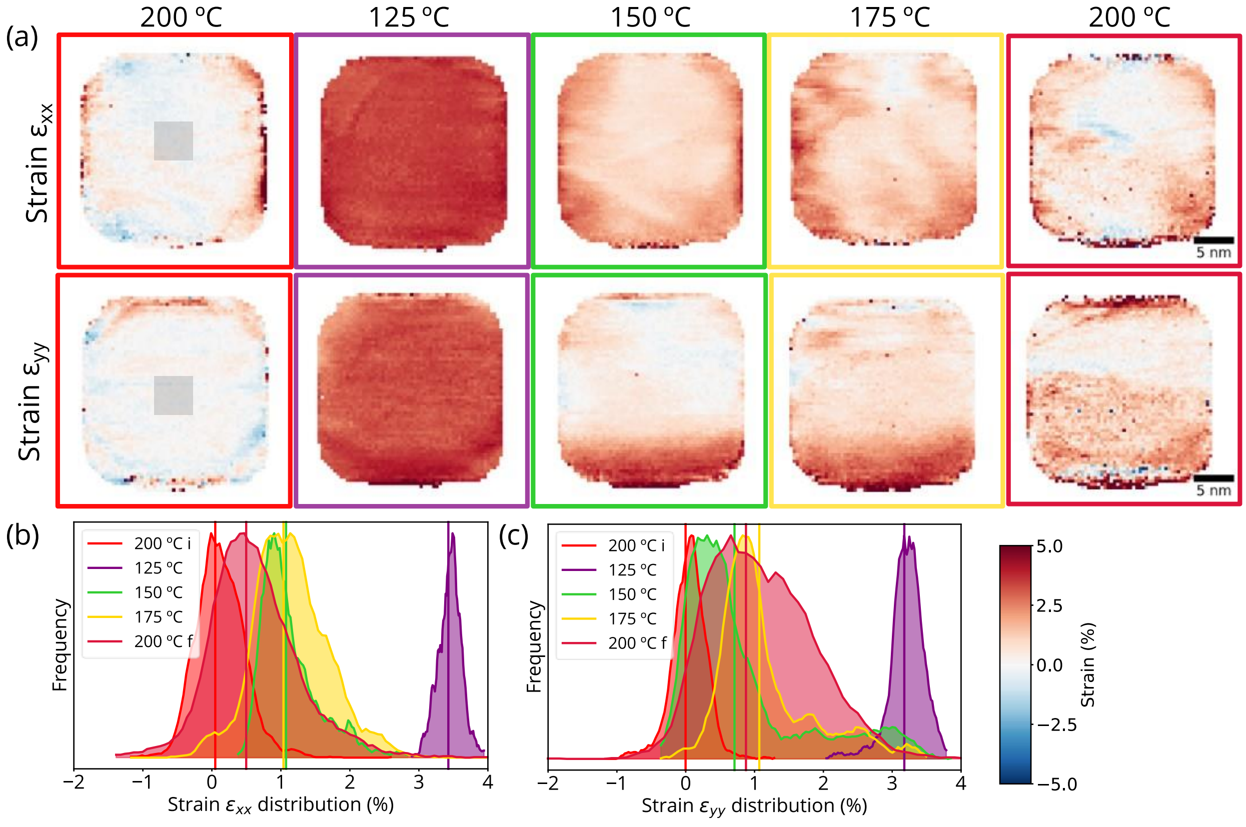

Figure S9. Strain mapping in 1 bar H_2, same nanoparticle as Figure 4 (a) Strain maps (x and y direction) obtained from 4D-STEM datasets using a region in the nanoparticle at 200 °C as reference (overlaid grey square in the 200 °C strain maps). The color scale is shown at the bottom with red and blue corresponding to tensile and compressive strain, respectively. (b) and (c) Normalized histogram of the strain distribution within a 10x10 nm region in the center of the nanoparticle at each temperature for strain in the x and y direction, respectively. The vertical lines correspond to the average strain at each temperature.

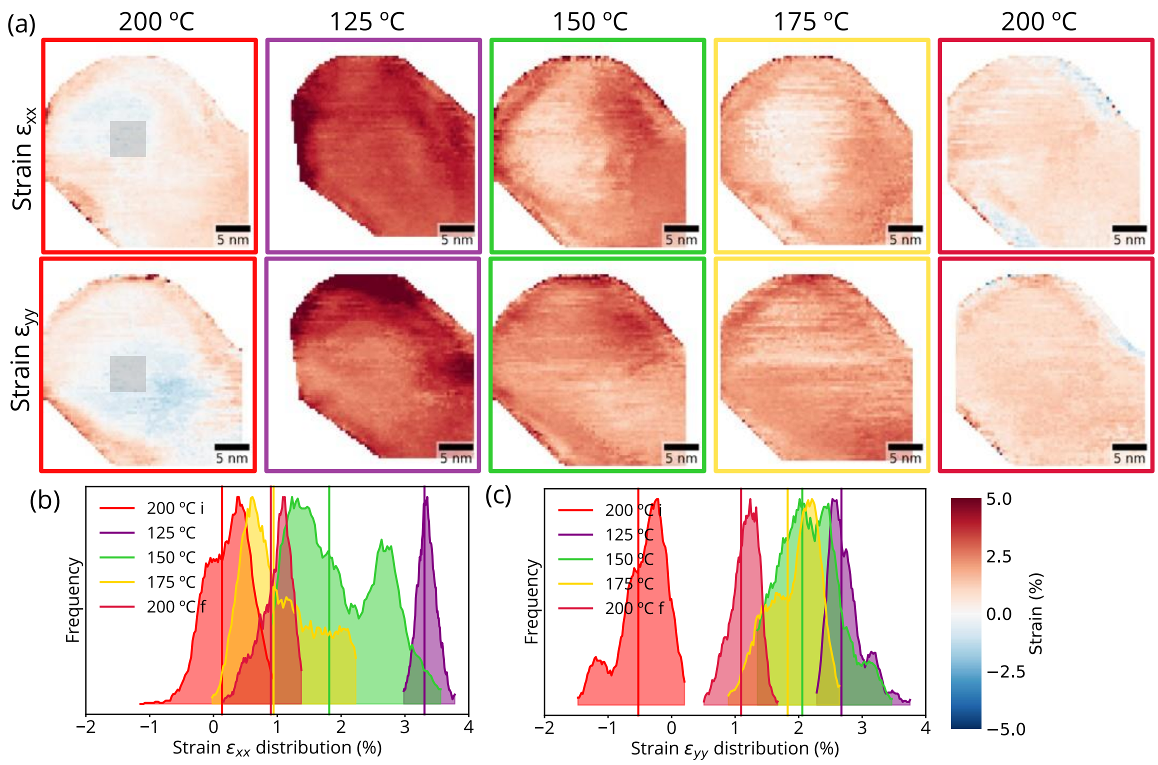

Figure S10. Strain mapping in 1 bar H_2, example 2 (a) Strain maps (x and y direction) obtained from 4D-STEM datasets using a region in the nanoparticle at 200 °C as reference (overlaid grey square in the 200 °C strain maps). The color scale is shown at the bottom with red and blue corresponding to tensile and compressive strain, respectively. (b) and (c) Normalized histogram of the strain distribution within a 10x10 nm region in the center of the nanoparticle at each temperature for strain in the x and y direction, respectively. The vertical lines correspond to the average strain at each temperature.

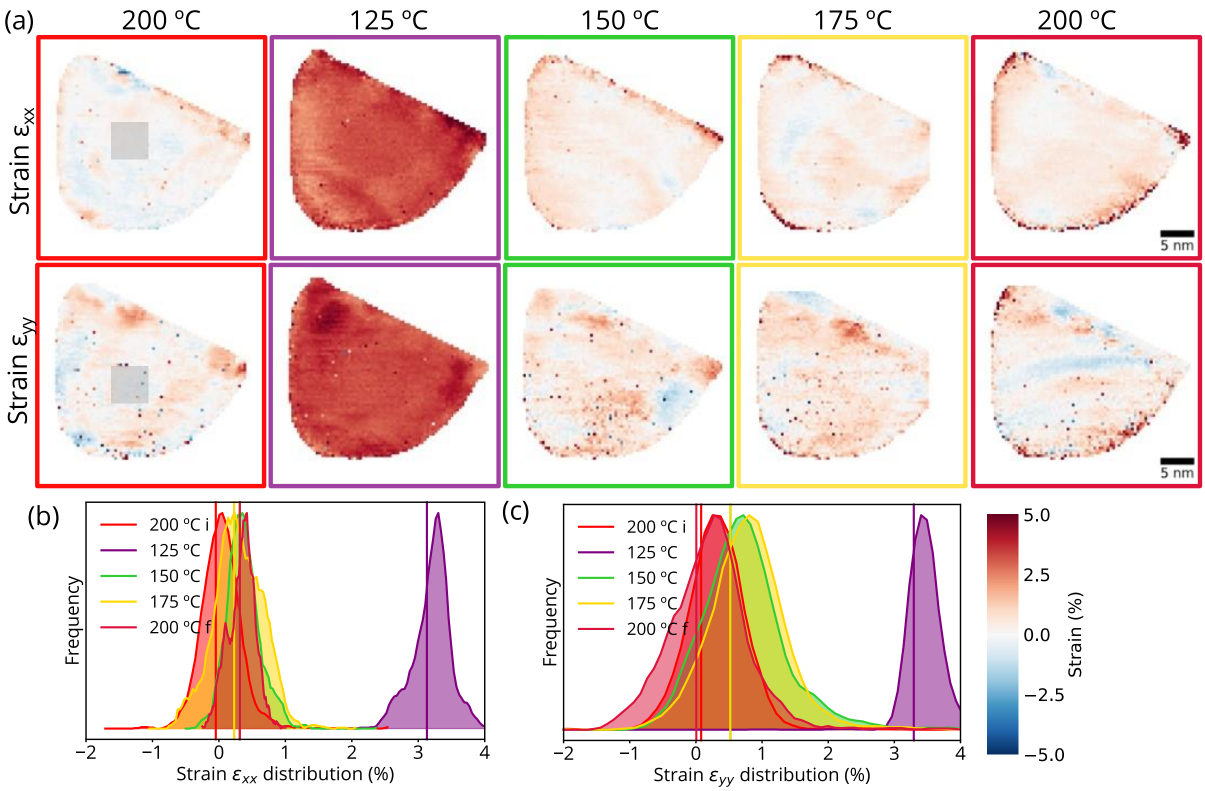

Figure S11. Strain mapping in 1 bar H_2, example 3 (a) Strain maps (x and y direction) obtained from 4D-STEM datasets using a region in the nanoparticle at 200 °C as reference (overlaid grey square in the 200 °C strain maps). The color scale is shown at the bottom with red and blue corresponding to tensile and compressive strain, respectively. (b) and (c) Normalized histogram of the strain distribution within a 10x10 nm region in the center of the nanoparticle at each temperature for strain in the x and y direction, respectively. The vertical lines correspond to the average strain at each temperature.

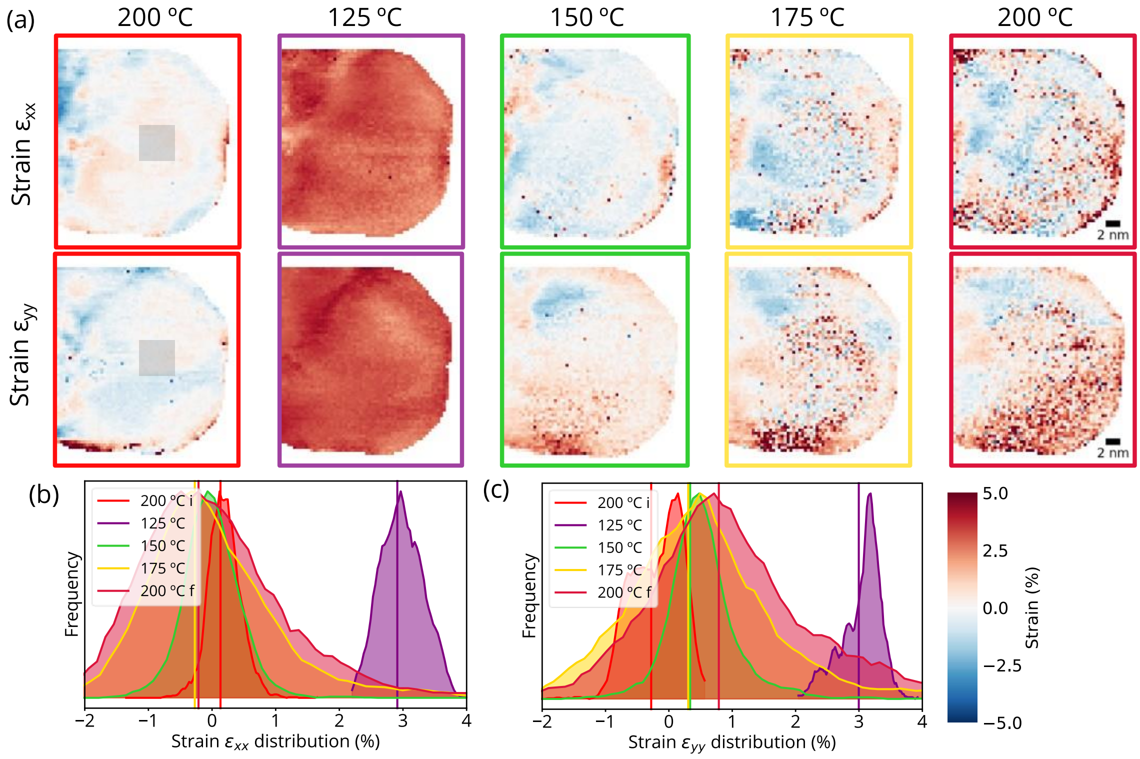

Figure S12. Strain mapping in 1 bar H_2, example 4. (a) Strain maps (x and y direction) obtained from 4D-STEM datasets using a region in the nanoparticle at 200 °C as reference (overlaid grey square in the 200 °C strain maps). The color scale is shown at the bottom with red and blue corresponding to tensile and compressive strain, respectively. (b) and (c) Normalized histogram of the strain distribution within a 10x10 nm region in the center of the nanoparticle at each temperature for strain in the x and y direction, respectively. The vertical lines correspond to the average strain at each temperature. Note that the strain maps above 150 °C are noisy, which is due to carbon contamination that decreased the contrast in the NBED patterns.

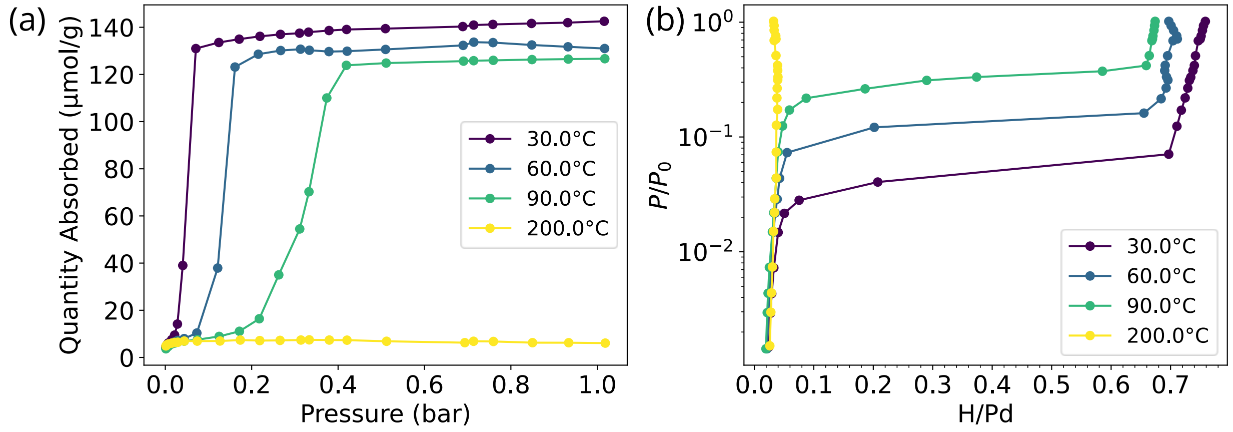

Figure S13. Temperature dependent hydrogen sorption capacity of Pd/SiO_2 nanocubes. (a) Hydrogen isotherms with an equilibration time of 10 s, measured at 30 °C (purple), 60 °C (blue), 90 °C (green) and 200 °C (yellow). (b) Pressure/composition isotherms obtained at 30 °C (purple), 60 °C (blue), 90 °C (green) and 200 °C (yellow). P_0 is 1 bar H_2.

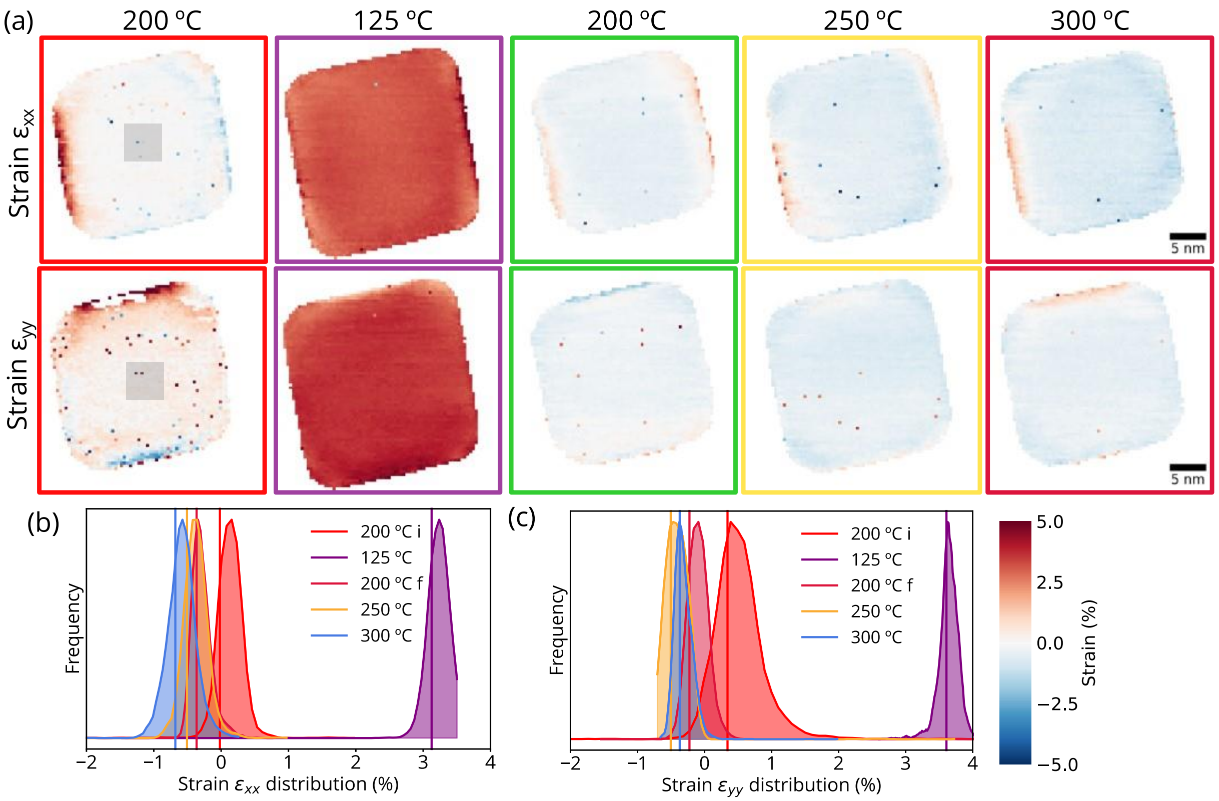

Figure S14. Strain mapping in 1 bar H_2 with heating up to 300 °C, example 1. (a) Strain maps (x and y direction) obtained from 4D-STEM datasets using a region in the nanoparticle at 200 °C as reference (overlaid grey square in the 200 °C strain maps). The color scale is shown at the bottom with red and blue corresponding to tensile and compressive strain, respectively. (b) and (c) Normalized histogram of the strain distribution within a 10x10 nm region in the center of the nanoparticle at each temperature for strain in the x and y direction, respectively. The vertical lines correspond to the average strain at each temperature.

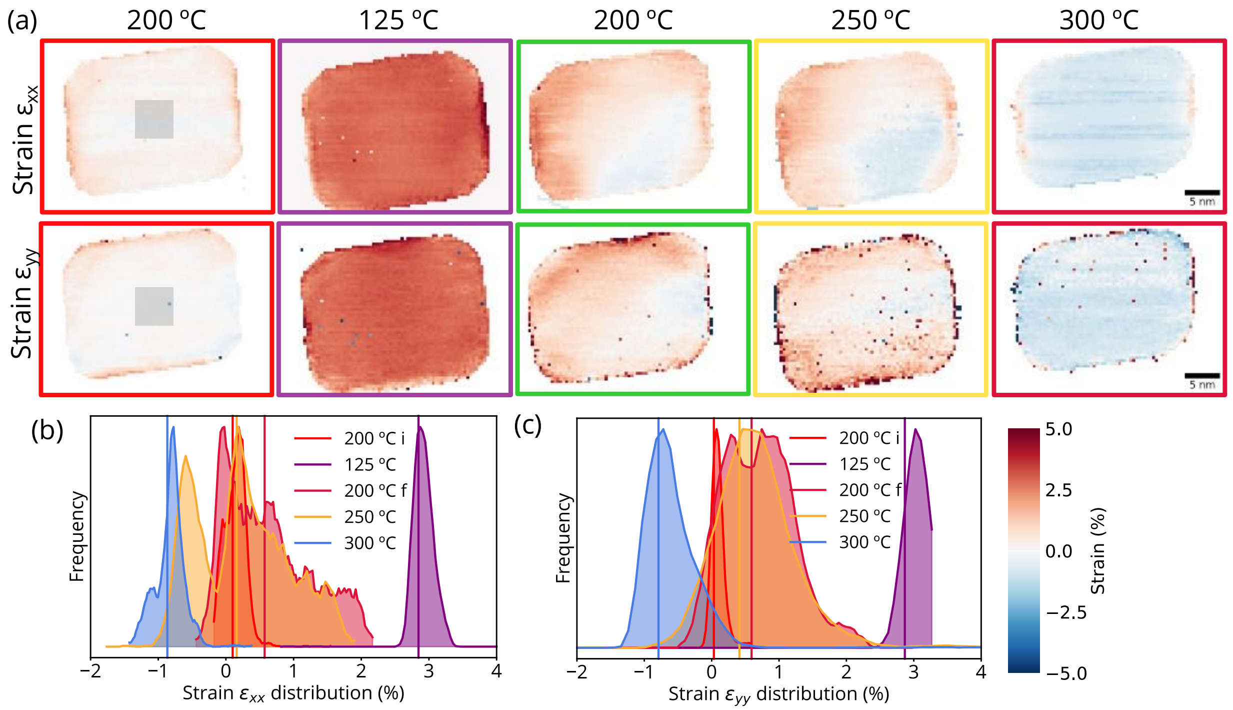

Figure S15. Strain mapping in 1 bar H_2, with heating up to 300 °C, example 2. (a) Strain maps (x and y direction) obtained from 4D-STEM datasets using a region in the nanoparticle at 200 °C as reference (overlaid grey square in the 200 °C strain maps). The color scale is shown at the bottom with red and blue corresponding to tensile sand compressive strain, respectively. (b) and (c) Normalized histogram of the strain distribution within a 10x10 nm region in the center of the nanoparticle at each temperature for strain in the x and y direction, respectively. The vertical lines correspond to the average strain at each temperature.

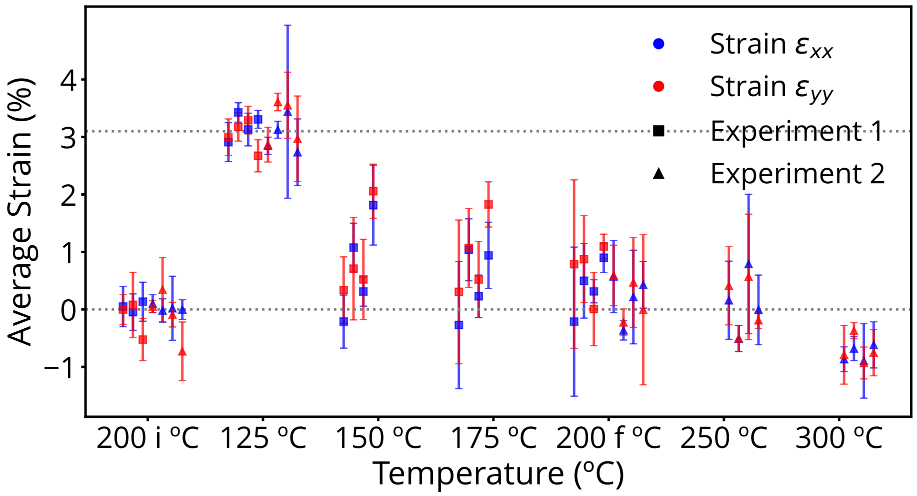

Figure S16 Summary of strain distribution at each nanoparticle at different temperatures. Each point represents the average strain ε_{xx} (blue) or ε_{yy} (red) in the histogram for one individual nanoparticle, with the errors being the standard deviation. The data with a squared marker is from the experiment 1, shown in Figure 4 and S9-12, and the data with the triangle marker is from experiment 2, shown in Figure S14-15. In each experiment, four nanoparticles were analysed.

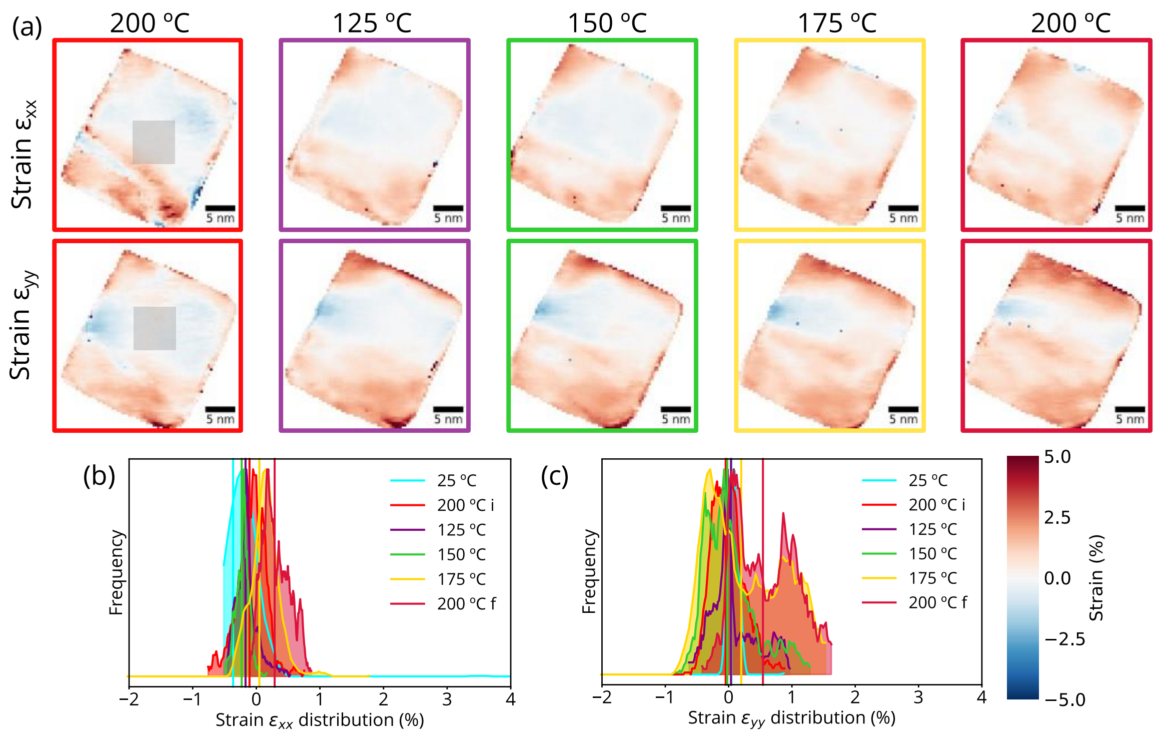

Figure S17. Strain mapping in vacuum at different temperatures, showing no significant difference in strain over the whole temperature range. (a) Strain maps (x and y direction) obtained from 4D-STEM datasets using a region in the nanoparticle at 200 °C as reference (overlaid grey square in the 200 initial strain maps). The color scale is shown at the bottom with red and blue corresponding to tensile and compressive strain, respectively. (b) and (c) Normalized histogram of the strain distribution within a 10x10 nm region in the center of the nanoparticle at each temperature, including room temperature, for strain in the x and y direction, respectively. The vertical lines correspond to the average strain at each temperature.

Supplementary note 1

Calculation of the H/Pd ratio from a lattice expansion.

A lattice expansion of 3.1 ± 0.3% would correspond to a lattice parameter of 403.1 ± 1.2 pm, calculated from the lattice parameter of the Pd nanocubes measured from the images in Figure 1b and 1c. The lineal increase upon increase of H/Pd ratio in the β-PdHx phase can be described by equation 1, as shown by Benck *et. al.,*[7] who corrected the obtained lattice parameters of several works for temperature and pressure.

Lattice parameter [pm] = 393.21 + 17.19 x H/Pd (Equation 1)

By substitution of the equation we find that the H/Pd ratio for such lattice expansion is of 0.58 ± 0.07.

Using a more traditionally used relation between the lattice parameter and H/Pd ratio (Equation 2)[8] yields that the H/Pd ratio in our nanoparticles is of 0.63 ± 0.10

Lattice parameter [pm] = 395.34 + 12.34 x H/Pd (Equation 2)

References

(1) Jin, M.; Liu, H.; Zhang, H.; Xie, Z.; Liu, J.; Xia, Y. Synthesis of Pd Nanocrystals Enclosed by {100} Facets and with Sizes <10 Nm for Application in CO Oxidation. *Nano Res.* **2011**, *4* (1), 83–91. https://doi.org/10.1007/s12274-010-0051-3.

(2) Liu, M.; Lyu, Z.; Zhang, Y.; Chen, R.; Xie, M.; Xia, Y. Atomic Layer-by-Layer Deposition of Pt on Pd Icosahedral Nanocrystals for Catalysts with Enhanced Activity and Durability toward Oxygen Reduction. *Nano Lett.* **2021**, *21* (5), 2248–2254. https://doi.org/10.1021/acs.nanolett.1c00007.

(3) Li, C.; Tardajos, A. P.; Wang, D.; Choukroun, D.; Van Daele, K.; Breugelmans, T.; Bals, S. A Simple Method to Clean Ligand Contamination on TEM Grids. *Ultramicroscopy* **2021**, *221* (December 2020), 113195. https://doi.org/10.1016/j.ultramic.2020.113195.

(4) Gatan. *4D STEM Strain Mapping*. https://www.gatan.com/4d-stem-strain-mapping (accessed 2024-10-11).

(5) Zlotea, C.; Oumellal, Y.; Provost, K.; Ghimbeu, C. M. Experimental Challenges in Studying Hydrogen Absorption in Ultrasmall Metal Nanoparticles. *Front. Energy Res.* **2016**, *4* (JUN), 1–11. https://doi.org/10.3389/fenrg.2016.00024.

(6) Liu, W.; Magnin, Y.; Förster, D.; Bourgon, J.; Len, T.; Morfin, F.; Piccolo, L.; Amara, H.; Zlotea, C. Size-Dependent Hydrogen Trapping in Palladium Nanoparticles. *J. Mater. Chem. A* **2021**, *9* (16), 10354–10363. https://doi.org/10.1039/D0TA12174F.

(7) Benck, J. D.; Jackson, A.; Young, D.; Rettenwander, D.; Chiang, Y. M. Producing High Concentrations of Hydrogen in Palladium via Electrochemical Insertion from Aqueous and Solid Electrolytes. *Chem. Mater.* **2019**, *31* (11), 4234–4245. https://doi.org/10.1021/acs.chemmater.9b01243.

(8) Manchester, F. D.; San-Martin, A.; Pitre, J. M. The H-Pd (Hydrogen-Palladium) System. *J. Phase Equilibria* **1994**, *15* (1), 62–83. https://doi.org/10.1007/BF02667685.
